# Laboratory-Based Factors Predicting Skiing Performance in Female and Male Biathletes

**DOI:** 10.3389/fspor.2020.00099

**Published:** 2020-08-05

**Authors:** Marko S. Laaksonen, Erik Andersson, Malin Jonsson Kårström, Hampus Lindblom, Kerry McGawley

**Affiliations:** Department of Health Sciences, Swedish Winter Sports Research Centre, Mid Sweden University, Östersund, Sweden

**Keywords:** aerobic, anaerobic, biathlon, gross efficiency, lactate threshold, maximal oxygen uptake, metabolic rate

## Abstract

Skiing in biathlon is a high-intensity, intermittent endurance discipline. This study aimed to evaluate the relationships between laboratory-derived physiological variables and skiing performance during a field-based biathlon competition (BC) for female and male biathletes. Fourteen female (23 ± 3 year, $\dot{V}$O_2max_ 56 ± 4 mL·kg^−1^·min^−1^) and 14 male (24 ± 4 year, $\dot{V}$O_2max_ 66 ± 3 mL·kg^−1^·min^−1^) biathletes performed a submaximal incremental test and a maximal time-trial (TT) using treadmill roller-skiing for the assessment of oxygen uptake at a lactate threshold of 4 mmol·L^−1^ ($\dot{V}$O_2@4mmol_), gross efficiency (GE), aerobic (MR_ae_) and anaerobic (MR_an_) metabolic rates, peak oxygen consumption ($\dot{V}$O_2peak_), anaerobic capacity and TT performance. Field-based skiing performance was assessed during a BC. The TT and BC skiing performances were significantly correlated in both sexes (*r* = 0.68–0.69, *p* < 0.01). $\dot{V}$O_2peak_ (31/21%), anaerobic capacity (1/0%), and GE (35/32%) explained 67 and 52% of the variance in BC skiing performance for the females (*p* < 0.01) and males (*p* = 0.051), respectively. A second model showed that $\dot{V}$O_2@4mmol_ (30/35%), anaerobic capacity (0/0%) and GE (37/13%) explained 67 and 48% of the variance in BC skiing performance for the females (*p* < 0.01) and males (*p* = 0.077), respectively. Results of this study suggest that a high $\dot{V}$O_2@4mmol_ and GE, but not anaerobic capacity, are important for BC skiing performance, especially for females. In addition, a laboratory-based TT could be useful for regular laboratory testing of biathletes due to its relationship with field-based skiing performance in biathlon.

## Introduction

Biathlon is an Olympic endurance sport combining cross-country skiing and rifle marksmanship. The length of traditional biathlon competitions are 6–15 km for females and 7.5–20 km for males, including two or four shooting bouts (International Biathlon Union, 2019b) each lasting approximately 25–35 s (Laaksonen et al., 2018b) and alternating between prone and standing positions. Since a biathlete completes three or five skiing bouts in varying terrain each lasting 5–8 min, the skiing component of biathlon is of a high-intensity, intermittent nature and makes the sport somewhat unique from a physiological perspective.

In endurance sports, maximal oxygen consumption ($\dot{V}$O_2max_) sets the upper limit for oxygen consumption ($\dot{V}$O_2_) during exercise, which is strongly associated with overall performance (Joyner and Coyle, 2008). In addition to a high $\dot{V}$O_2max_, a well-developed economy or efficiency of movement reduces the overall energy cost during exercise (Joyner and Coyle, 2008) and might be equally as important to overall performance, as has been observed in cross-country skiing (Sandbakk et al., 2010; Andersson et al., 2017). Studies of cross-country skiing have shown that $\dot{V}$O_2max_, the $\dot{V}$O_2_ at lactate threshold and economy of movement are related to skiing performance in distance competitions covering 10 km (Mahood et al., 2001; Carlsson et al., 2016). Rundell and Bacharach (1995) showed with national-level biathletes that peak $\dot{V}$O_2_ ($\dot{V}$O_2peak_) and $\dot{V}$O_2_ at the lactate threshold obtained during treadmill running exercise were associated with biathlon skiing performance in females, whereas no significant associations were found for the males. Recently, Jonsson Kårström et al. (2019) also showed that treadmill skiing speed at 4 mmol·L^−1^ of blood lactate concentration correlated strongly to performance during an ~ 3-min time-trial. Furthermore, the average heart rate (HR) while skiing during a biathlon competition is high, at ~ 90% of HR_max_ (Hoffman and Street, 1992).

In addition to aerobic parameters, anaerobic energy delivery also plays an important role in high-intensity endurance performance. For example, work intensities over 100% of $\dot{V}$O_2max_ have been observed in uphill sections during cross-country skiing (Norman et al., 1989; Andersson et al., 2017; Karlsson et al., 2018). Moreover, laboratory-based cross-country skiing sprint race simulations lasting ~3–4 min suggest that the relative anaerobic energy contribution is ~ 17–26% (Losnegard et al., 2012; McGawley and Holmberg, 2014; Andersson et al., 2017). However, no previous studies appear to have investigated the relationships of different physiological capacities derived in the laboratory during treadmill roller-skiing with field-based skiing performance in biathlon.

Unlike in the sport of cross-country skiing, biathletes are required to carry a rifle weighing ≥ 3.5 kg on their backs during competition. Despite this clear distinction from cross-country skiing, which significantly affects the physiological demands of the sport (Rundell and Szmedra, 1998; Stöggl et al., 2015; Jonsson Kårström et al., 2019), skiing performance in biathlon has been sparsely studied. It has been suggested that skiing performance explains approximately 59–65% (Luchsinger et al., 2018; Dzhilkibaeva et al., 2019) and 42–54% (Luchsinger et al., 2019) of the variation in performance in World-cup biathlon sprint and individual competitions, respectively, while shooting accuracy and shooting time explain the remainder of the variation (Luchsinger et al., 2018, 2019; Dzhilkibaeva et al., 2019). Rundell (1995) observed almost 25 years ago moderate to strong correlations between roller-skiing performance and both $\dot{V}$O_2peak_ and $\dot{V}$O_2_ at the lactate threshold based on seven female biathletes. However, the skiing speed during biathlon sprint competitions has increased during the last decade (Laaksonen et al., 2018a) and, moreover, the associations between field-based skiing performance and both anaerobic energy metabolism and gross efficiency (GE) have not previously been evaluated in biathlon. Furthermore, only one study (Rundell and Bacharach, 1995) appears to have investigated the sex differences associated with field-based skiing performance in biathlon. This is despite studies in cross-country skiing showing differences in $\dot{V}$O_2peak_ (Hegge et al., 2016) but similarities in GE (Sandbakk et al., 2013a; Hegge et al., 2016) for males vs. females.

Therefore, the aim of the present study was to investigate the relationships between field-based biathlon skiing performance and a range of laboratory-derived variables determined from submaximal and maximal roller-skiing tests on a treadmill, including $\dot{V}$O_2peak_, the $\dot{V}$O_2_ at a lactate concentration of 4 mmol·L^−1^ ($\dot{V}$O_2@4mmol_), aerobic (MR_ae_) and anaerobic (MR_an_) metabolic rates, GE and time-trial (TT) performance, in females and males. This knowledge may provide biathlon coaches with valuable information for planning training programs.

## Materials and Methods

### Participants

Fourteen female (age 19–29 year) and 14 male (age 19–32 year) Swedish biathletes, including junior and senior Biathlon World Championship medalists and several top-15 ranked athletes in Biathlon World Cup competitions, were recruited to the study (Table 1). Four females and three males had not participated in International Biathlon Union (IBU) competitions and were therefore missing IBU points. Fifteen of the participants were members of the Swedish Biathlon Federation's national, developmental or junior squads and 10 further participants belonged to the Mid Sweden University elite biathlon program. All participants had several years of experience in endurance training and roller-skiing (both in the field and on a treadmill) and had completed 11–15 h·week^−1^ of endurance training on average per year. Participants gave their written consent after being informed of the purpose, nature, benefits and potential risks involved prior to initiation of the tests. The study was pre-approved by the Regional Ethical Review Board of Umeå University, Umeå, Sweden (#2016-506-31M) and was conducted according to the Declaration of Helsinki.

**Table 1:** Mean ± SD characteristics and statistical comparisons of the female and male biathletes.

	**Females**	**Males**	***p*-value**	***ES***
Age (years)	22.9 ± 3.2	24.0 ± 3.9	0.437	0.30
Height (cm)	166.4 ± 6.3	178.8 ± 6.2	<0.001	1.97
Body mass (kg)	62.8 ± 5.6	74.8 ± 5.6	<0.001	2.15
IBU points	81.6 ± 38.3	76.3 ± 36.2	0.745	0.14
	(*n* = 10)	(*n* = 11)		

*ES, effect size; IBU points, International Biathlon Union ranking points, where lower points imply a higher performance level*.

### Data Collection and Analysis

Female and male biathletes completed a submaximal incremental test and a maximal self-paced TT on a laboratory treadmill during pre-season (within a 2-week period in September). Data from the laboratory tests were used to assess $\dot{V}$O_2@4mmol_, metabolic rates, GE, $\dot{V}$O_2peak_ and TT performance. Within 2 months of the laboratory testing (in November) all participants performed a field-based biathlon competition (BC), where skiing performance was assessed as total BC time minus shooting time.

### Laboratory Tests

The laboratory tests were conducted on a motor-driven treadmill (Rodby Innovation AB, Vänge, Sweden) using Pro-Ski S2 roller skis (Sterners, Dala-Järna, Sweden) equipped with NNN (Rottefella, Klockarstua, Norway) or SNS (Salomon, Annecy, France) bindings. The roller skis were pre-warmed for at least 60 min in a heating box before all tests to control for variations in rolling resistance (Ainegren et al., 2008). The rolling resistance (μ_R_) was determined as described previously (Ainegren et al., 2008) and average μ_R_ was calculated as 0.0224 and 0.0216 for the NNN and SNS binding systems, respectively. Only the G3 skating sub-technique (symmetrical poling with simultaneous leg work) was used and participants wore a safety harness around their waist, which was suspended from the ceiling and connected to an emergency brake. After reporting to the laboratory, a standardized 6-min warm-up was performed at 7 km·h^−1^ and a gradient of 3.5° for the females, and at 8 km·h^−1^ with a gradient of 4.5° for the males. After warming up participants performed the submaximal incremental test, which consisted of 3–5 stages each lasting 4 min, which started at the same inclination and speed as the warm-up intensity and increased by 2 km·h^−1^ per stage thereafter. The submaximal test was used to identify $\dot{V}$O_2@4mmol_ and ceased when the following criteria were met: RER > 1.0, ${\dot{V}}_{\text{E}}\cdot \dot{V}$ ${\text{O}}_{2}^{-1}$ > 30, HR ≥ 90% of known HR_max_ and a rating of perceived exertion (RPE) of ≥ 16 out of 20.

The submaximal test was followed by 4 min of active recovery at warm-up pace, 5 min of passive recovery and a 5-min active re-warm-up including three, 10–15 s self-paced intervals at perceived race pace. The automated self-pacing system built into the treadmill detected the position of the participant via a laser system and increased (0.68 km·h^−1^·s^−1^) or decreased (0.40 km·h^−1^·s^−1^) the speed of the treadmill if the participant moved to the front or rear of the treadmill, respectively (Swarén et al., 2013). The self-paced TT was performed after the 5-min re-warm-up and was 900 m for the females (inclination 3.5°, starting speed 13 km·h^−1^) and 1,000 m for the males (inclination 4.5°, starting speed 14 km·h^−1^). The average relative power output (W·kg^−1^) during the TT test was used to compare TT performance between the females and males.

During both the submaximal and TT tests, respiratory variables were measured using an AMIS 2001 model C ergospirometry system (Innovision A/S, Odense, Denmark) with 10-s sampling periods. Gas analyzers were calibrated with a mixture of 16.0% O_2_ and 4.5% CO_2_ (Strandmöllen AB, Ljungby, Sweden) and calibration of the flowmeter was performed at low, medium, and high flow rates with a 3-L air syringe (Hans Rodolph, Kansas City, Missouri, USA). During the submaximal test, $\dot{V}$O_2_, $\dot{V}$CO_2_ and respiratory exchange ratio (RER) were determined during the last 30 s of each stage. $\dot{V}$O_2@4mmol_ was determined from the exponential relationship between $\dot{V}$O_2_ and blood lactate concentration (B-La). During the TT, the highest average of three consecutive 10-s $\dot{V}$O_2_ values was defined as $\dot{V}$O_2peak_.

Fingertip blood samples (20 μL) were taken immediately after each submaximal workload and 2 min after the TT for the determination of B-La using a Biosen S-line (EKF diagnostic GmbH, Magdeburg, Germany), which was calibrated with a standard solution of lactate (12 mmol·L^−1^) prior to each analysis. The HR was monitored with a V800 apparatus (Polar Electro Oy, Kempele, Finland) and ambient conditions were monitored with an external apparatus (Vaisala PTU 200, Vaisala Oy, Helsinki, Finland).

### Biathlon Competition

Within 2 months of completing the laboratory tests, all participants competed in the same field-based biathlon competition (BC). Weather conditions were stable, with air temperature and wind speed averaging 0.7°C and 1.8 m·s^−1^, respectively, and no precipitation. The competition was an open individual race organized by the Swedish Biathlon Federation and formed the basis of the coming season's selection for the international biathlon teams. The total number of participants in the competition was 31 females and 41 males. The competition was a modification of a typical individual race and included five skiing bouts of 2 km for the females (total climbing: 69 m per lap) or 2.5 km for the males (total climbing: 75 m per lap), thus the total course length was 10 km for females and 12.5 km for males. There were four shooting bouts (prone, standing, prone, standing), including five shots per bout, where each missed shot resulted in a 40-s time penalty. The start list was randomly generated for all competitors and the starting interval between all athletes was 30 s. During BC, the total skiing time (including time in the range but excluding shooting time) was determined and converted to an average skiing speed, which was used to reflect skiing performance in the correlational analyses. IBU points were obtained from the IBU datacenter (International Biathlon Union, 2019a) for athletes participating in international competitions (i.e., World cup, World Championships, IBU cup and/or IBU Open European Championships), where lower IBU points reflect a higher ranking.

### Calculations

All calculations were based on those reported by Andersson et al. (2017). Specifically, the power output (PO), MR_ae_, MR_an_ and GE were determined individually at the submaximal workload where RER was closest to 1.00, but in all cases ≤ 1.00. PO was calculated according to Equation (1):

(1) $$\begin{array}{l} PO\;\left[W\right]=\;vm_{sys}\left(g\;\sin\left(\alpha \right)+u_{R}g\;\cos\left(\alpha \right)\right) \end{array}$$

where *g* reflects gravitational acceleration, *v* the speed of the treadmill, μ_R_ the rolling resistance, and α the inclination of the treadmill. The MR_ae_ was calculated from $\dot{V}$O_2_ (L·min^−1^) and RER values ≤ 1.00 as follows:

(2) $$MR_{ae}\;[W]=\;\frac{4184(\dot{V}O_{2}(1.1RER+3.9))}{60}$$

The GE was calculated as:

(3) $$\begin{array}{l} GE\left(\%\right)=100\left(PO÷\;MR_{ae}\right) \end{array}$$

The average PO during the TT (PO_tt_) was calculated as described in Equation (1), using the average TT speed. The required metabolic rate (MR_req_) during the TT was calculated as PO_tt_ divided by GE and converted to energy required relative to body mass (E_req_). The average MR_ae_ during the TT was determined by using the average $\dot{V}$O_2_ during the TT and Equation (2) (assuming 100% carbohydrate utilization, i.e., using an RER value of 1.00), and MR_an_ was subsequently calculated as MR_req_ minus MR_ae_. The total aerobic energy contribution (E_ae_) in Joules relative to body mass during the TT was calculated by integrating MR_ae_ over TT time, then dividing by body mass. Finally, anaerobic energy contribution (E_an_), calculated as E_req_ minus E_ae_, was converted to an accumulated O_2_ deficit (AOD) by multiplying the E_an_ with a constant of 0.047801 (ml O_2_ equivalent·J^−1^) according to Weir (1949) and assuming 100% carbohydrate utilization during the supramaximal TT.

### Statistical Analysis

All statistical tests were performed with SPSS statistical software (release 18.0.0, SPSS Inc., Chicago, IL) using an alpha level of ≤ 0.05. The normal distribution of all parameters was tested using a Shapiro-Wilks test together with inspection of qq-plots and histograms. When comparing the sexes, two-tailed Student *t*-tests for independent samples were used. Comparisons of GE, PO_tt_ and metabolic rates (during the TT) between sexes were not appropriate due to the use of different protocols for females and males. Pearson's product-moment analyses were used to calculate bivariate correlation coefficients. A block-wise multiple regression analysis was used to analyze how the variation in the laboratory-derived variables explained the variation in BC skiing performance. The magnitude of differences between sexes were expressed as standardized mean differences (Cohen's *d* effect size; ES), where the criteria for interpreting the magnitudes of ES were < 0.2 trivial, 0.2–0.6 small, 0.6–1.2 moderate, 1.2–2.0 large, and > 2.0 very large (Hopkins et al., 2009). All data are expressed as mean ± standard deviation (SD).

## Results

The overall performance and physiological responses during the submaximal and maximal TT tests are presented in Table 2. The $\dot{V}$O_2@4mmol_ during the submaximal test was significantly higher for males than females. In addition, the $\dot{V}$O_2peak_, ${\dot{V}}_{\text{Emax}}$ and maximal B-La derived from the TT were higher for males compared to females, while the AOD only tended to be higher in males (*p* = 0.096).

**Table 2:** Mean ± SD performance and physiological responses during the submaximal and maximal time-trial (TT) tests.

**Submaximal test**	**Females (3.5^**°**^)**	**Males (4.5^**°**^)**	***p*-value**	**ES**	**Interpretation**
$\dot{V}$O_2@4*mmol*_ (mL·kg^−1^·min^−1^)	46.1 ± 4.6	54.9 ± 3.6	<0.001	2.13	Very large
$\dot{V}$O_2@4*mmol*_ (% of $\dot{V}$O_2*max*_)	82.1 ± 3.6	81.3 ± 3.9	0.610	0.20	Small
GE (%)	16.6 ± 0.8	18.4 ± 1.1			
**Maximal TT test**	**Females 900 m (3.5°)**	**Males 1,000 m (4.5°)**			
TT time (s)	190 ± 13	198 ± 12			
Average speed (m·s^−1^)	4.8 ± 0.3	5.1 ± 0.3			
PO_tt_ (W·kg^−1^)	3.9 ± 0.3	5.0 ± 0.3			
$\dot{V}$O_2*max*_ (mL·kg^−1^·min^−1^)	55.6 ± 4.1	66.0 ± 3.2	<0.001	2.86	Very large
HR_max_ (beats·min^−1^)	188 ± 6	191 ± 7	0.339	0.37	Small
V_Emax_ (L·min^−1^)	136 ± 16	192 ± 17	<0.001	3.36	Very large
B-La_max_ (mmol·L^−1^)	12.4 ± 2.3	14.1 ± 1.6	0.031	0.88	Moderate
MR_req_ (W·kg^−1^)	23.5 ± 1.5	27.3 ± 1.4			
MR_ae_ (W·kg^−1^)	18.1 ± 1.1	21.2 ± 1.1			
MR_an_ (W·kg^−1^)	5.4 ± 1.1	6.1 ± 1.7			
E_ae_ (%)	77.0 ± 3.8	77.8 ± 5.5			
E_an_ (%)	23.0 ± 3.8	22.2 ± 5.5			
AOD (mL·kg^−1^)	49.2 ± 9.9	57.7 ± 15.5	0.096	0.67	Moderate

*ES, effect size; $\dot{V}$O_2@4mmol_, oxygen consumption at a lactate concentration of 4 mmol·L^−1^; GE, gross efficiency; PO_tt_, power output during the TT; $\dot{V}$O_2max_, maximal oxygen consumption; HR, heart rate; V_Emax_, maximal ventilation; B-La_max_, maximal blood lactate concentration; MR_req_, required metabolic rate; MR_ae_, aerobic metabolic rate; MR_an_, anaerobic metabolic rate; E_ae_, aerobic energy contribution; E_an_, anaerobic energy contribution; AOD, accumulated oxygen deficit*.

**Figure 1:**
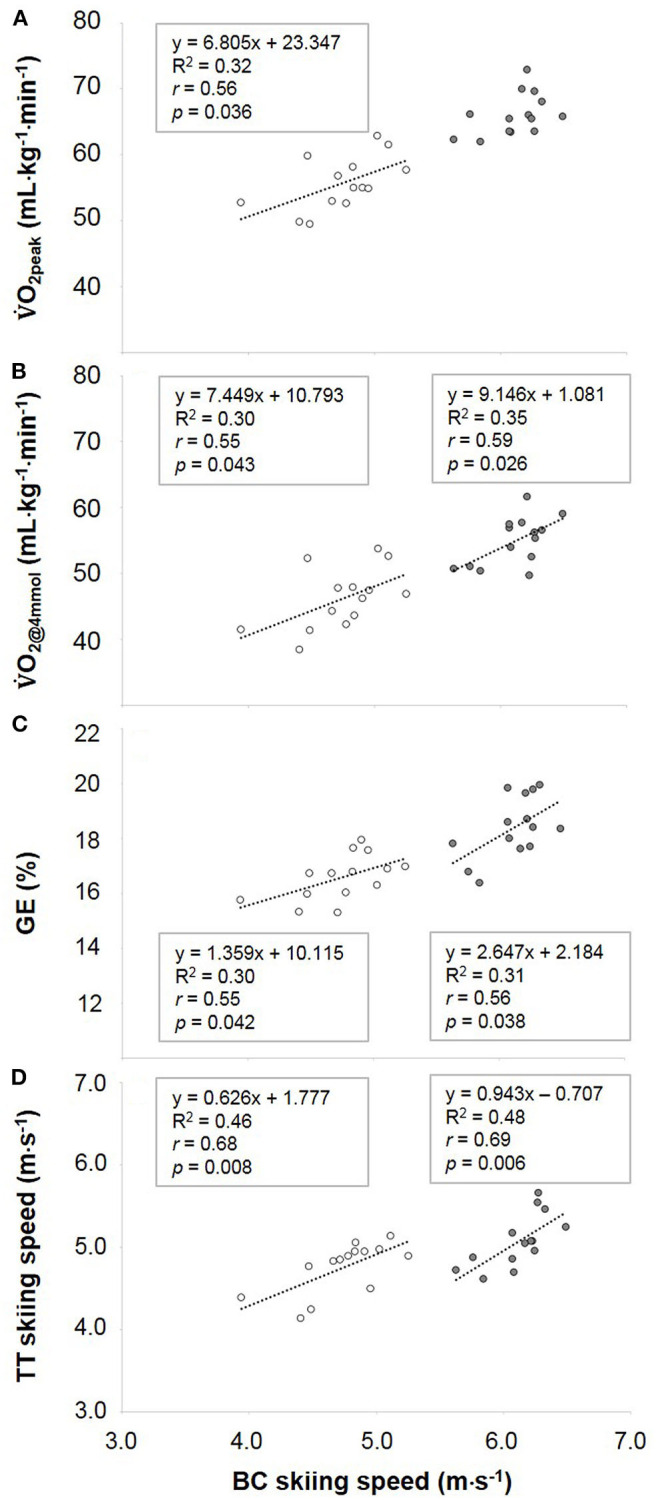
Linear associations between biathlon competition (BC) skiing speeds and **(A)** peak oxygen consumption ($\dot{V}$O_2peak_), **(B)** oxygen consumption at a lactate concentration of 4 mmol/L ($\dot{V}$O_2@4mmol_), **(C)** gross efficiency (GE) and **(D)** time-trial (TT) skiing speeds for females (unfilled circles) and males (filled circles).

During BC, the average skiing speed was higher for males than for females (6.1 ± 0.2 m·s^−1^ vs. 4.7 ± 0.3 m·s^−1^; *p* < 0.001). $\dot{V}$O_2peak_ was correlated to BC skiing performance in females but not males (Figure 1A). $\dot{V}$O_2@4mmol_ and GE were significantly correlated to BC skiing performance in both sexes (Figures 1B,C), whereas MR_ae_, MR_an_ and AOD were not correlated to BC skiing performance for either sex (Table 3). TT performance was significantly correlated to BC skiing performance in both sexes (Figure 1D). With BC skiing performance as the dependent variable and $\dot{V}$O_2peak_, E_an_ and GE as independent variables, a block-wise multiple regression analysis resulted in a significant *R*^2^-value for females (*R*^2^ = 0.67, *p* < 0.01), where the three variables explained 31, 1, and 35% of the variation, respectively. For males, the *R*^2^-value tended to be significant (*R*^2^ = 0.52, *p* = 0.051), with $\dot{V}$O_2peak_, E_an_ and GE explaining 21, 0, and 32% of the variation in BC skiing performance, respectively. When $\dot{V}$O_2@4mmol_ replaced $\dot{V}$O_2peak_ in this regression model, the *R*^2^-value was significant for females (*R*^2^ = 0.67, *p* < 0.01) where $\dot{V}$O_2@4mmol_, E_an_ and GE explained 30, 0, and 37% of the variation in BC skiing performance, respectively. For the males, the *R*^2^-value only tended to be significant (*R*^2^ = 0.48, *p* = 0.077) where $\dot{V}$O_2@4mmol_, E_an_ and GE explained 35, 0, and 13% of the variation in BC skiing performance.

**Table 3:** Pearson product-moment correlation coefficients (*r*) for biathlon competition (BC) skiing speed (performance) and selected laboratory-derived variables.

	**BC skiing speed**	
	**Females**	**Males**
$\dot{V}$O_2@4mmol_(% of $\dot{V}O_{2\text{peak}}$)	0.39	0.11
MR_ae_ (W·kg^−1^)	0.42	0.36
MR_an_ (W·kg^−1^)	−0.05	−0.09
AOD (mL·kg^−1^)	−0.03	−0.28

*$\dot{V}$O_2@4mmol_, oxygen consumption at a lactate concentration of 4 mmol·L^−1^; MR_ae_, aerobic metabolic rate; MR_an_, anaerobic metabolic rate; AOD, accumulated oxygen deficit*.

## Discussion

The present study aimed to evaluate how different laboratory-derived variables relate to field-based biathlon skiing performance in female and male biathletes. The main finding was that $\dot{V}$O_2@4mmol_ and GE were the most important factors for BC skiing performance in both sexes. In addition, TT performance was significantly correlated to BC skiing performance.

Females demonstrated ~16% lower absolute $\dot{V}$O_2@4mmol_ and $\dot{V}$O_2peak_ values compared to males. These findings are consistent with earlier reports in cross-country skiing (Sandbakk et al., 2012b) and have been partly attributed to lower body fat and a higher hemoglobin concentration and stroke volume in males (Joyner, 1993). In general, the $\dot{V}$O_2peak_ values were rather low in our study population compared to a previous biathlon study by Tønnessen et al. (2015), as well as earlier studies in cross-country skiing (Sandbakk et al., 2010, 2012b; Losnegard et al., 2012; Losnegard and Hallén, 2014) investigating elite athletes employing the same sub-technique (i.e., G3 skating). This may be due to the likely wider range of performance levels among the biathletes recruited to the present study, from a World Cup winner to national-level competitive athletes.

The GE was comparable to values recently presented for well-trained biathletes (Jonsson Kårström et al., 2019), but was slightly higher than values shown for cross-country skiers using the G3 skating sub-technique (Sandbakk et al., 2010, 2012a, 2013a; Ainegren et al., 2013). This difference is likely due to the different population groups studied and/or the use of different test protocols or equipment for measuring respiratory variables. While in the present study males demonstrated a higher GE compared to females, although not statistically compared, earlier investigations in cross-country skiing have reported similar GE values between sexes (Sandbakk et al., 2012b, 2013a; Hegge et al., 2016). Possible explanations for these discrepancies between studies might be different work rates and inclines used during the submaximal tests (Sandbakk et al., 2012a), the performance level of the athletes (Sandbakk et al., 2013a) and/or the level of technical skill during roller-skiing. Indeed, Pellegrini et al. (2017) recently demonstrated that better technical skills during double poling are related to an enhanced skiing economy. The AOD was not significantly different between sexes in the present study, although the group means demonstrated a tendency for the males to elicit higher values. McGawley and Holmberg (2014) showed similar results regarding AOD in junior cross-country skiers using diagonal skiing (i.e., a tendency for a higher AOD among males).

When using bivariate correlation analyses, $\dot{V}$O_2@4mmol_ and GE were both significantly related to BC skiing performance in both sexes. Using multiple regression analysis, $\dot{V}$O_2@4mmol_ and GE both explained more than 30% of the variation in BC skiing performance for females. For males, only $\dot{V}$O_2@4mmol_ was significant in the model. This finding supports earlier investigations demonstrating the importance of $\dot{V}$O_2@4mmol_ and GE for endurance performance (Joyner and Coyle, 2008). Thus, developing these variables would appear to be important for improving skiing performance in biathlon. A significant correlation was also identified between $\dot{V}$O_2peak_ and BC skiing performance for females, whereas the same correlation did not reach significance for the males. Using multiple regression analysis, $\dot{V}$O_2peak_ explained ~ 32% of the variation in BC skiing performance for females but only 21% for males. As $\dot{V}$O_2peak_ is one of the main physiological determinants of endurance performance (Joyner and Coyle, 2008) it is likely that this difference between sexes is partly related to the considerably higher between-athlete variation in both $\dot{V}$O_2peak_ and BC skiing performance (as seen in Figure 1) among the females compared to the males in the present study.

It has been demonstrated that work intensity during uphill cross-country skiing can exceed the level of $\dot{V}$O_2peak_ (Norman et al., 1989; Andersson et al., 2017; Karlsson et al., 2018). Given the undulating terrain in biathlon, and the intermittent nature of the sport, the anaerobic component may also be considered important for biathlon skiing performance. However, neither MR_an_ nor AOD were associated with BC skiing performance in the present study. This is in contrast to the findings of Losnegard and colleagues, who found a moderate correlation between AOD and skiing performance during a short, ~ 180 s self-paced roller-skiing TT using the same sub-technique as in the present study (Losnegard et al., 2012). Although the terrain in the BC was relatively variable in the present study, the duration of each lap was rather long (~7 min for both the females and males), indicating a more dominant aerobic component. However, a high day-to-day variation in anaerobic capacity (Watkins et al., 2017) as well as the known uncertainties in the determination of anaerobic capacity (Noordhof et al., 2010) could also potentially limit any significant association to BC skiing performance.

The TT performance was significantly correlated to BC skiing performance for both sexes. This suggests that the TT test utilized in the present study may be useful in reflecting skiing performance in a field-based BC, despite the total duration of the endurance efforts differing substantially. Recently, McGawley (2017) suggested that a TT provides more reliable performance data than an incremental test to exhaustion. Moreover, previous laboratory-based cross-country skiing studies have shown that similar or even higher $\dot{V}$O_2peak_ values can be achieved during a self-paced TT compared to traditional incremental tests (McGawley and Holmberg, 2014; Andersson et al., 2017). Therefore, 3-min laboratory-based TT tests, as used in the present study, may be more ecologically valid to assess both performance and $\dot{V}$O_2peak_ in well-trained athletes.

It should be acknowledged that the laboratory tests were completed nearly 2 months before the BC. During that time period fitness levels may have changed, although it is likely that potential variations in performance, as well as in aerobic and anaerobic capacities, would have been rather small. This is supported by the findings of Losnegard et al. (2013), who observed no significant changes in $\dot{V}$O_2peak_, AOD or TT performance during a similar training period (i.e., pre-season) among elite cross-country skiers. An additional source of measurement error in field-based studies is the risk of changing weather conditions and in ski-based studies, specifically, the changing physical properties of the snow, which can have a meaningful impact on the kinetic friction between the ski base and snow surface. However, during the BC there was no snow- or rainfall and the temperature and wind speed were stable throughout the competition, which lasted ~ 45 min in total (from the first start to the last finish). In addition, all athletes used comparable ski-base preparations (i.e., similar stone grinds and glide wax) performed by professional ski technicians. Therefore, it is likely that external weather factors, different ski-base preparations and selected skis for racing would only have affected BC skiing performance to a small extent in the current study. In addition, while the significant *R*^2^-values between the laboratory-derived variables and BC skiing performance were moderate, it must be acknowledged that biathlon skiing performance, as in other endurance sports, is also affected by biomechanical and psychological factors which were not considered in the present study. For example, the laboratory test was performed with roller skiing using only skating G3 sub-technique whereas the BC skiing in the field on snow includes additional sub-techniques. Therefore, future studies should also consider the use of other sub-techniques in laboratory testing.

From a methodological perspective, the most frequently used sub-technique during biathlon competions (i.e., G3) was chosen for the submaximal laboratory testing, as well as different treadmill speeds and inclines for the females and males. A steeper slope for the females would have resulted in an inability to apply the chosen sub-technique and/or a significantly higher exercise intensity than the males. Slowing down the treadmill speed in that case would have resulted in a greater difference in speed between the sexes. In contrast, if the males had been prescribed the same slope as the females then the exercise intensities during the lower sub-maximal stages would have been too easy for them. These differences between testing protocols for the females and males prevented some comparisons between sexes.

Over the last decades the skiing speeds in biathlon, similar to skating in cross-country skiing, have increased markedly (Sandbakk and Holmberg, 2014; Laaksonen et al., 2018a,b; International Biathlon Union, 2019a). Therefore, it is of interest to update the scientific knowledge of the physiological capacities that are important for performance, in order to inform training practices. According to the present results, a high $\dot{V}$O_2@4mmol_ and GE have a major impact on skiing performance in biathlon for both males and females and it would therefore be advisable to encourage training regimens that focus on improving these capacities. However, it cannot be excluded that a high $\dot{V}$O_2peak_ is needed for superior skiing performance, which was shown for the female biathletes in the present study. From a practical perspective, high-intensity interval training has been recommended for improving $\dot{V}$O_2max_ and performance (see e.g., Buchheit and Laursen, 2013), which may also be beneficial for improving lactate threshold (Sandbakk et al., 2013b). In addition, resistance and/or plyometric training, explosive resistance training and training at near-maximal or supramaximal intensities might be beneficial for skiing economy as has been shown for running (Barnes and Kilding, 2015). However, since biathlon has been sparsely studied in the past, further investigations involving trained biathletes, especially field-based studies, are required to provide a more thorough scientific basis to the practices adopted by coaches, athletes and practitioners.

In conclusion, this is the first study to our knowledge that has investigated how different laboratory-derived variables during roller skiing, including aerobic and anaerobic metabolic rates and gross efficiency, are related to skiing performance during a real-world, field-based biathlon competition over varying terrain with elite-level biathletes. According to the present results, a high $\dot{V}$O_2@4mmol_ and GE are of major importance for BC skiing performance, especially for females, while the role of anaerobic capacity for BC skiing performance remains unclear. Field-based biathlon skiing performance is affected by a wide variety of complex, interacting factors, and may not therefore be entirely explained by laboratory-derived variables. Thus, changes in environmental conditions and/or conducting the BC on a more flat or demanding course profile, compared to the present study, may result in different associations between laboratory-derived variables and skiing performance in the field. However, the TT test used in the current study appears to be a useful marker of field-based skiing performance in biathlon.

## Data Availability Statement

The datasets generated for this study are available on request to the corresponding author.

## Ethics Statement

The studies involving human participants were reviewed and approved by Regional Ethical Review Board of Umeå University, Umeå, Sweden. The patients/participants provided their written informed consent to participate in this study.

## Author Contributions

ML contributed to research concept and study design. ML, EA, and KM contributed to literature review. ML and HL contributed to data collection. ML, EA, MJ, HL, and KM contributed to data analysis and interpretation. ML and EA performed the statistical analysis. ML, EA, MJ, HL, and KM wrote and edited the manuscript as well as approved the final version to be published and agreed to be accountable for all aspects of the work. All authors contributed to the article and approved the submitted version.

## Conflict of Interest

The authors declare that the research was conducted in the absence of any commercial or financial relationships that could be construed as a potential conflict of interest.
